# Sporopollenin Surface Characterization with Inverse Liquid Chromatography

**DOI:** 10.1021/acsomega.5c07819

**Published:** 2025-12-08

**Authors:** Oluwatimilehin O. Fadiran, J. Carson Meredith

**Affiliations:** School of Chemical and Biomolecular Engineering, 1372 Georgia Institute of Technology, 311 Ferst Drive, NW, Atlanta, Georgia 30032, United States

## Abstract

Pollen grains are microscopic particles with unique surface chemistries tuned by evolution to protect delicate genetic material. The exine is composed of a cross-linked material, sporopollenin, which is known to have a high modulus and to be highly resistant to chemical and oxidative degradation. Developing a quantitative understanding of sporopollenin surface chemistry and its interactions with other molecules is relevant to understanding sporopollenin’s natural function as well as applications as an engineering material. By utilizing ragweed (*A. artemisiifolia*) pollen spores as a fixed phase, we describe a novel use of inverse liquid chromatography to probe the interactions of sporopollenin with organic compounds of varying polarity. By measuring capacity factors of probes at various temperatures, heats of interaction were calculated. Retention behavior of defatted (D) and acid–base treated (AB) pollen were compared. D pollen displayed low capacity factors that were nearly the same for most probes, except for ethanol, the smallest and most polar probe. On the other hand AB pollen displayed capacity factors that depended strongly on the polarity of the chemical probe. Chemical probes with alcohol and amine groups were highly retained on AB-treated pollen. Notably, the capacity factor, peak asymmetry, and enthalpy of interaction passed through a maximum as a function of probe polarity, and enthalpy ranged from −9 kJ/mol (benzene) to −41 kJ/mol (pyridine). These results suggest that the surface of the AB pollen is polar and likely protic with strong Lewis acid characteristics.

## Graphical abstract

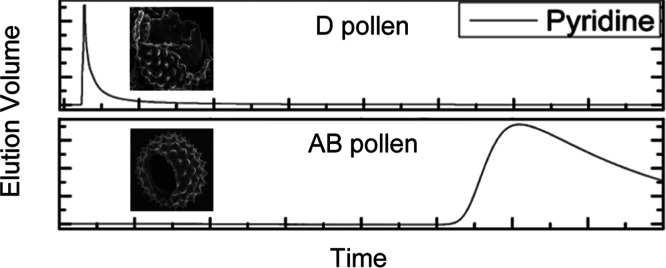

## Introduction

Pollen grains are microscopic particles exhibiting complex surface chemistries and solid surface features, which protect delicate internal genetic material crucial for plant reproduction and which also facilitate adhesion of pollen to insect and plant surfaces under different dynamic and environmental conditions.^1^ The pollen wall has three main domains: the solid intine and exine, and a lipid-rich liquid external pollen coat.^2,3^ The outermost exine is composed of sporopollenin, a cross-linked copolymer with an aliphatic core and oxygenated aromatic functional side groups.^1,3–8^ Figure 1 presents one proposed sporopollenin structure that synthesizes many features of prior literature, which we adopt here as a framework for understanding the aliphatic, aromatic, ether, acid, and hydroxyl functional units that are general features observed in many studies. More recent structural studies reveal additional details about the chemistry of sporopollenin. For example, Li et al. utilized NMR spectroscopy to characterize pine sporopollenin. They showed that it possesses aliphatic-polyketide-derived poly­(vinyl alcohol) units and coumaroyl-modified aliphatic units that are cross-linked through a dioxane/acetal group.^6^

**Figure 1:**
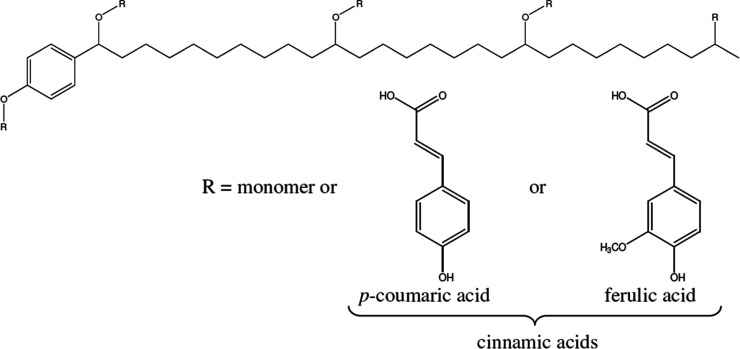
General structure of sporopollenin and monomeric units. Used with permission from Barrier.^1^

There are only limited quantitative studies on the role of pollen surface chemistry in the surface interactions of pollen grains with other substances. A quantitative description of the interactions of pollen with organic compounds bearing different functional groups is useful for further understanding pollen interactions in the natural environment, as well as in engineering applications. For example, our group has explored the use of pollen in polymer composites^9–11^ and in the creation of inorganic replicas.^12^ Pollen have also been used to form microgels^13^ and to encapsulate pharmaceuticals and vaccines for human delivery.^14–16^ An important feature in these applications is the significant influence of pollen extraction methods. Defatting is a common treatment involving washing with organic solvents, e.g., diethyl ether or acetone, to remove lipids and other surface contaminants.^17^ A further treatment of D pollen by base hydrolysis removes internal cellular hydrolyzable organics, and another treatment with warm phosphoric acid removes the cellulose intine. The resulting acid–base (AB) treated pollen has only the sporopollenin exine shell intact. This is thought to make surface sites more readily available for chemical modification and adsorption.^3,18^ For example, both D and AB pollen have been used as mechanical-reinforcing lightweight fillers for epoxy, where it was found that AB treatment was essential for observing mechanical property improvement.^9^ Similar findings were reported for the use of AB pollen as a reinforcing filler in composites with poly­(vinyl acetate) (PVAc).^10^ Further, surface-treated AB pollen have been used as templates to form complex metal-oxide and metallic replicas through conformal coating with organometallic precursors.^12^

Inverse liquid chromatography (ILC) can be used to study solid–liquid interactions and is sensitive to small differences in adsorption properties through the measurement of retention times of different chemical probes. ILC is similar to inverse gas chromatography (IGC), a technique that has been used over the past 50 years,^19–22^ with the key difference being that probes are delivered in the liquid state. One ILC approach is based on the determination of the capacity factors of chemical probes from their net retention times at near infinite dilution.^23–26^ ILC retention may be due to multiple mechanisms, including van der Waals interactions, hydrogen bonding, and size exclusion. Solid–liquid interactions may be influenced by several factors, such as the functional groups of the chemical probes and on the solid surface, their distribution on the surface, and surface area and porosity. By use of probes with a variety of functional groups and polarity, characteristics of the solid surface relevant to chemical interactions can be determined. For example, ILC has been used to determine the physicochemical characteristics of inorganic and organic solids including silica, coal, xerogels, and zirconia.^27–29^ A complication with ILC is that the use of liquid components results in competition between the mobile phase and probe molecules for adsorption in the solid phase. To minimize competition, often a nonpolar, well-characterized solvent is used as a mobile phase when exploring interactions between solids and more polar molecules. On the other hand, column efficiency is less significant in ILC than in analytical high-performance liquid chromatography (HPLC), where peak resolution and efficient separation is the goal.

In the present study, ILC was used to determine the capacity factors of a series of organic compounds of varying polarity in columns packed with ragweed (*A. artemisiifolia*) pollen grains. Both D and AB pollen were examined in order to better understand the accessibility and interactions of the pollen material before and after cleaning. We hypothesize that sporopollenin will have enhanced interactions with molecules that have Lewis acid and base characteristics, but it is unclear whether an acid or base will be preferred. Cyclohexane was used as the mobile phase, and the chemical probes included aromatic (benzene), ketone (acetone), ester (ethyl acetate), protic alcohol (ethanol and 2-propanol), and amine (pyridine) compounds. By measuring capacity factors at different temperatures, the enthalpy of interaction of each probe with the AB pollen surface was determined. The dependence of probe capacity factor, peak asymmetry, and enthalpy of interaction on probe physicochemical properties was determined, including dipole moment, dielectric constant, kinetic diameter, and Hildebrand solubility parameter. This analysis revealed the potential for sporopollenin to undergo strong chemical interactions with Lewis bases and alcohols. To the best of our knowledge, this is the first use of ILC in characterizing intact pollen grains.

## Results and Discussion

Figure 2 shows scanning electron microscopy (SEM) images of crushed D and AB pollen, which reveal significant differences in structure. In D pollen, we observe evidence for intracellular material on the interior of the pollen exine as well as material that fills in the porous spaces in the exine wall. Intracellular material includes storage oil bodies, membrane phospholipids, and pollen cytoplasm.^2^ In contrast, AB pollen is hollow and free of an intercellular material. In addition, the interior of the exine wall appears porous, free of extraneous material, and the AB treatment has revealed the fine 30 to 100 nm pores on the outer exine. The specific surface areas of D and AB pollen determined by the BET method were 1.95 m^2^/g (D) and 5.80 m^2^/g (AB), indicating an increase in the surface area of AB compared to D pollen, in alignment with the SEM results.

**Figure 2:**
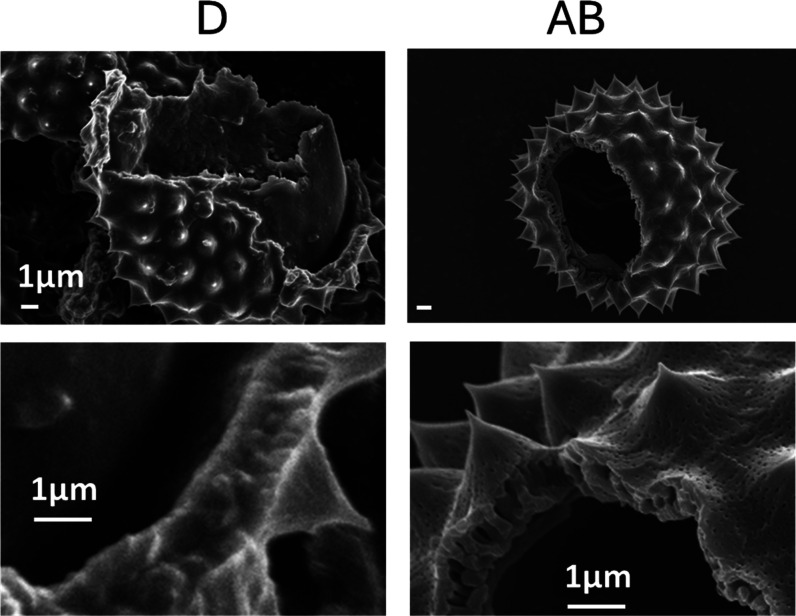
SEM of crushed D and AB ragweed pollen at two magnifications, revealing residual intracellular and cellulosic intine material in D. AB pollen is hollow with intracellular material and cellulosic intine removed.

Figure 3 shows FTIR spectra for both the D and AB pollen. The spectra of the different pollen bear some similarity but exhibit key differences. Numerous peaks between 1200 cm^–1^ and 1600 cm^–1^ are present in both D and AB pollen, with two exceptions at 1540 cm^–1^ (hemicellulose)^30^ and 1303 cm^–1^ (cellulose CH_2_ wag),^31^ which are absent in AB. Present in both samples are peaks at 1428 cm^–1^ (CH_2_ bending), 1403 cm^–1^ (CH_2_ bending), and 1368 cm^–1^ (CH bend).^31^ The band at 1540 cm^–1^ has been observed in hemicellulose^31^ and is possibly associated with carboxylate stretching,^32^ but it is absent in AB pollen. The band at 1518 cm^–1^ in D pollen, likely representing protein content (amide II),^33^ is effectively absent in AB pollen, although a very small peak remains at 1514 cm^–1^ in AB pollen. The 1514 cm^–1^ band has been attributed to phenyl ring vibrations in sporopollenin.^33^ Two peaks present as shoulders in D pollen are resolved into isolated peaks in AB pollen, which are the skeletal aromatic C–C band at 1578 cm^–1^ emanating from sporopollenin,^16,33^ and the band at 1232 cm^–1^. The latter band can indicate either the C–OH OH bending in cellulose or an aromatic C=O stretching,^31^ which would be expected in the sporopollenin exine. Fingerprint bands associated with hemicellulose^30^ and cellulose^5,34^ ether bonds appear in the 1100 cm^–1^ to 800 cm^–1^ region on D pollen, with peaks at 997 cm^–1^ (−C–O–C– ring stretch) and 856 cm^–1^ (β-glycoside –C–O–C– stretch) due to the presence of the intine polysaccharide material. The peaks appear to be shifted to a lower wavenumber than in pristine cellulose (the β-glycoside peak usually shows at 895 cm^–1^),^30^ which may be associated with their mixture with other substances or association with sporopollenin. Nevertheless, in the AB pollen spectrum, these peaks are significantly diminished (997 cm^–1^) or lacking altogether (856 cm^–1^), indicating the full or partial dissolution of pollen cellulosic intine.^1,7^ D pollen also shows a broad set of bands between 600 and 700 cm^–1^, most likely associated with an –OH bend in cellulose, which is significantly diminished in AB pollen.^33^ Additionally, D pollen displays a very broad band at 3375 cm^–1^ due to the O–H stretch of −OH groups that shows a stronger intensity than the −CH stretches of −CH_2_ groups at 2910 and 2840 cm^–1^. In contrast, AB pollen displays a more narrow −OH band with a smaller intensity than the −CH_2_ peaks. AB pollen’s OH band is also shifted to higher wavenumbers around 3450 cm^–1^, indicating increased free hydroxyls. The C=O stretch of carboxyl groups also shifts from 1672 to 1707 cm^–1^ on D and AB pollen, respectively. Finally, the bands at 1630 cm^–1^ (D) and 1650 cm^–1^ (AB) are likely associated with bending vibrations of absorbed water and cellulose –OH groups, respectively.^30^ The spectra support the full or partial dissolution of the intine in AB pollen that would make the hydroxyl and carboxyl groups more accessible for surface interactions.

**Figure 3:**
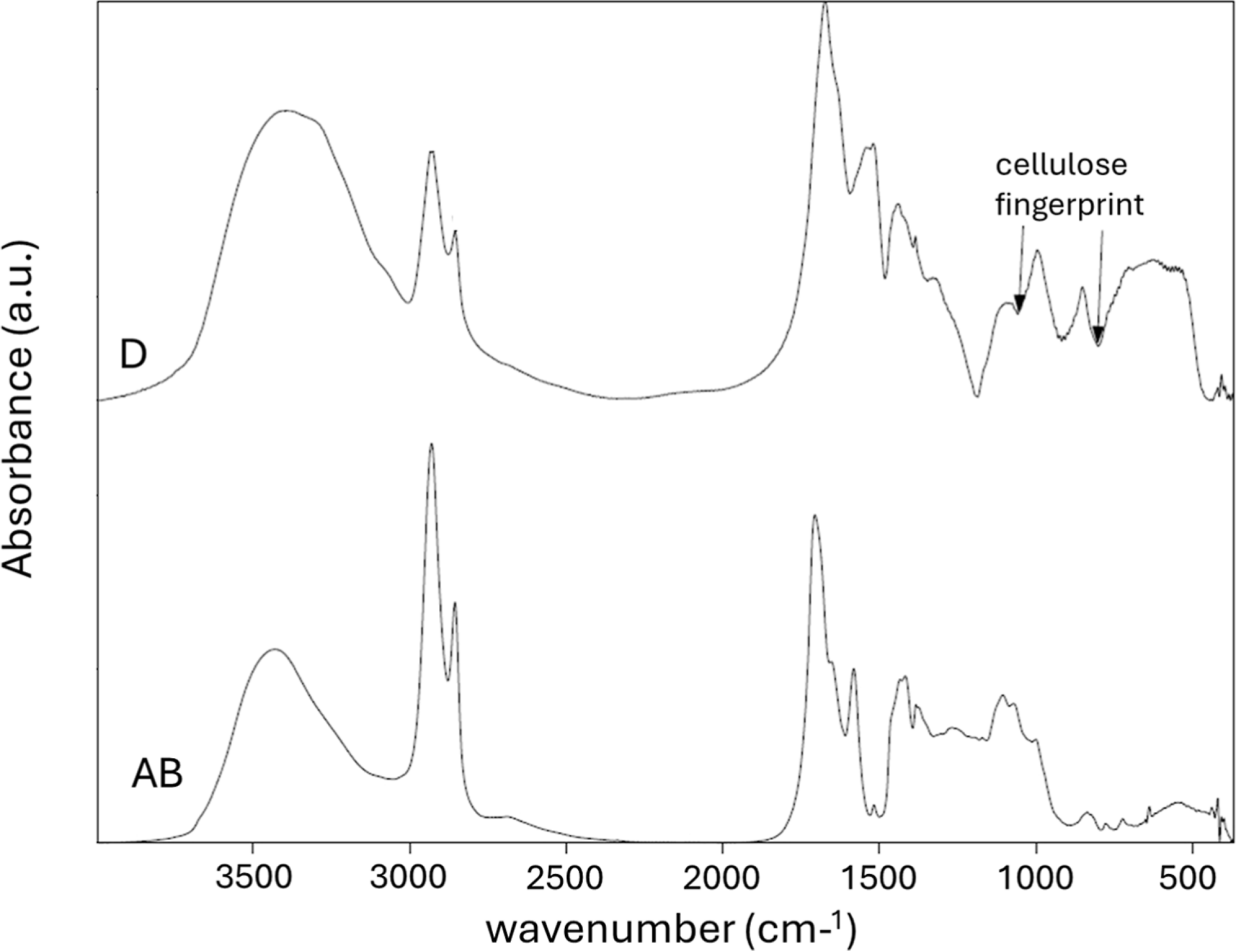
FTIR spectra of D and AB pollen in arbitrary units (a.u.) of absorbance.

Residuals of phosphoric acid treatment can remain on sporopollenin due to being trapped within exine pores and binding of phosphate ions to hydroxyl or phenolic groups on sporopollenin. If residuals are present, they are expected to occur as phosphates due to the neutralization step used after the acid treatment. We do not detect the presence of typical P–O (900–1100 cm^–1^) or P=O (1150–1250 cm^–1^) bands in FTIR,^35^ although we cannot rule out trace residuals due to the sensitivity of FTIR. The P–O asymmetric stretching between 1000 and 1100 cm^–1^ unfortunately can overlap significantly with C–O–C bands that may be present on sporopollenin. However, we do not observe the P–O symmetric peak between 900 and 950 cm^–1^.^35^ If residual phosphate or phosphoric acid groups are present in trace quantities, then they would be expected to mimic some of the behavior of –C=O and –COOH groups on AB pollen. FTIR results above suggest that hydroxyl and groups oxygenated carbon dominate the polar components of the AB sporopollenin structure.

Figure 4a shows characteristic chromatograms obtained at 30 °C for molecular probes on the D pollen. With the exception of ethanol, it is observed that the peak retention time is very similar for all probes, regardless of polarity (i.e., dielectric constant, dipole moment, ability to hydrogen bond, etc.), falling near 1.4 min. In contrast to the behavior of other probes, ethanol eluted at 4.26 min. Table S1 shows the *A*_S_ values for the probes on the D-pollen at three different temperatures. While ideal Gaussian *A*_S_ ∼ 1 is not a requirement for ILC, as it is for efficient analytical HPLC, it can indicate strong interactions between the solid and liquid phase probes.^36^ The solvent cyclohexane had *A*_S_ values of 1.67–1.72, which may arise from nonideal elution associated with the reduced accessibility of the surface indicated by SEM (Figure 2). Many of the probes, including the carrier solvent, have similar *A*_S_ values between 1 and 2. *A*_S_ increases with probe polarity, particularly for acetone, ethanol, propanol, and pyridine. The results for elution time, taken together with the SEM, surface area, and FTIR results above, suggest that the intine and other material present in the D-pollen exine, not removed by defatting, blocks the access to the sporopollenin surface to most probes. The increased capacity factor for ethanol may be related to its small size relative to the other probes (Table 1), combined with its polarity and its ability for participating in hydrogen bonds. Ethanol is the smallest probe and has the highest dielectric constant and solubility parameter, the latter of which likely drives its high retention in D pollen from the nonpolar carrier solvent.

**Figure 4:**
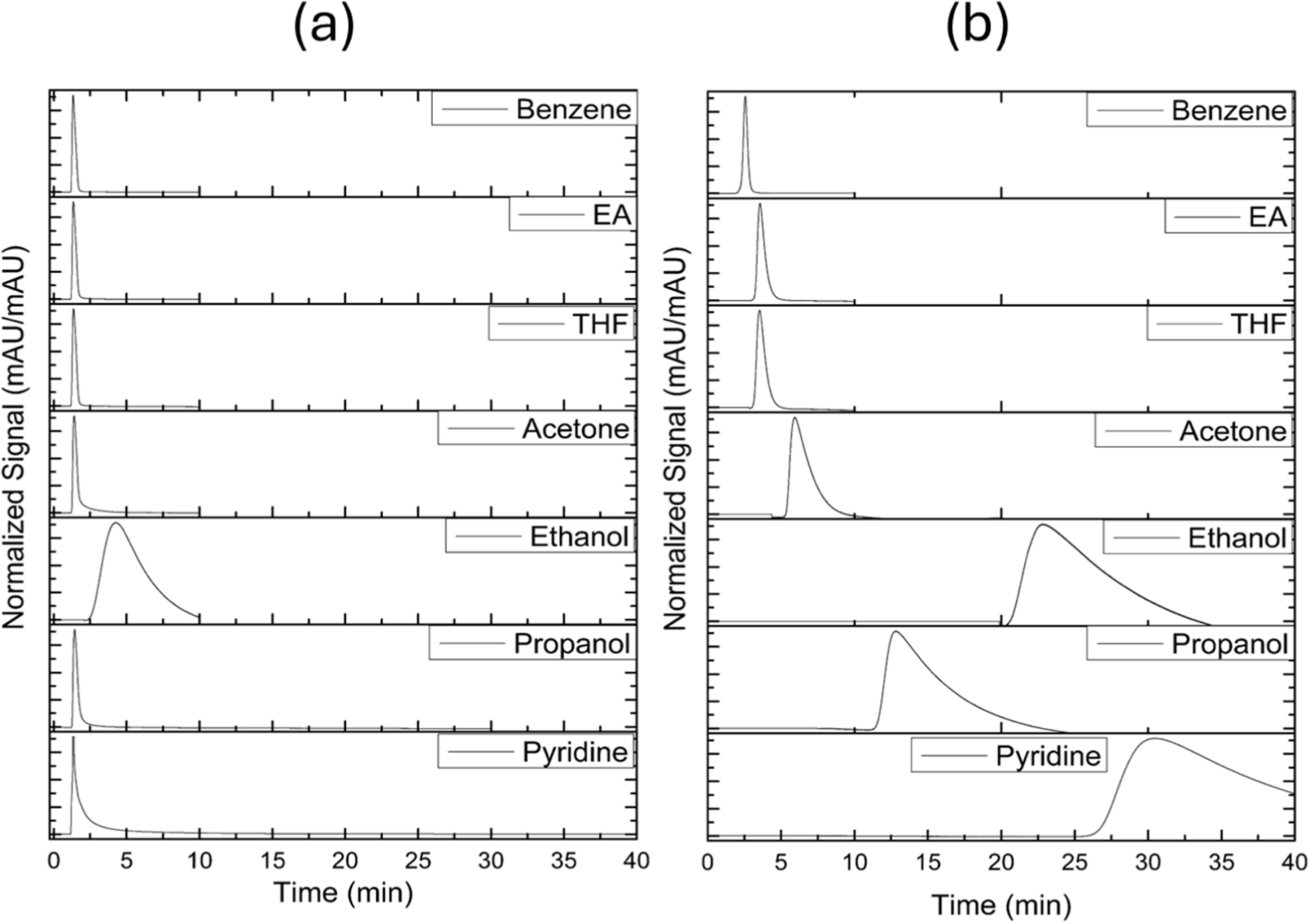
Characteristic chromatograms for retention on (a) D pollen at 30 °C and (b) AB pollen at 50 °C. EA is ethyl acetate and THF is tetrahydrofuran.

**Table 1:** Properties of Chemical Probes and the Mobile Phase (Cyclohexane)

probe	kinetic diameter (Å)	dielectric constant, ε	dipole moment, μ (D)	solubility parameter, δ (MPa^1/2^)
acetone	5.0	20.7	2.88	19.7
benzene	5.9	2.27	0	18.7
cyclohexane	6.0	2.02	0	16.8
ethanol	4.5	24.5	1.68	26.2
ethyl acetate	5.5	6.02	1.78	18.2
isopropanol	4.8	17.9	1.58	24.9
pyridine	5.0	12.4	2.19	21.7
tetrahydrofuran	5.1	7.58	1.63	18.5

Figure 4b shows the representative retention chromatograms for AB pollen at 50 °C, and Table 2 shows the AB pollen capacity factors for the probes at three different temperatures. Peak asymmetry values are given in Table S2 in the Supporting Information. AB pollen displayed a retention behavior significantly different from that of D pollen. Benzene had the lowest capacity factor of 1.07 at 30 °C, eluting in 2.8 min. Oxygen-containing aprotic molecules were more highly retained. Ethyl acetate and tetrahydrofuran showed similar capacity factors of 2.6 and 2.5 at 30 °C, respectively, while acetone was more highly retained with a capacity factor of 5.9. Alcohols propanol and ethanol showed still higher capacity factors of 17 and 33, respectively. Pyridine had the largest elution time, 70 min at 30 °C, with a corresponding capacity factor of 50, far exceeding all other probes. As expected, capacity factors decrease with increased temperature, while the general trends of capacity factor with the probe molecular structure presented above remains the same at all temperatures. Whitman et al. reported *k* values for a wide variety of probes on acid-washed zirconia eluting from 99% hexane/1% chloroform.^37^ The trends in *k* with solvent type mirror the results obtained here, going from low to high *k* values in the order benzene, acetone, alcohols, and pyridine. Pollen particles, with their ornate surface morphology and potential for generation of debris during cleaning, have not been used as a solid phase in HPLC to our knowledge. It is worth noting that these features could introduce irregular packing that might affect the retention reproducibility or peak asymmetry. As detailed in the Experimental Methods section, we took precautions during AB pollen preparation to isolate intact pollen grains free of debris. In addition, we used identical vacuum-assisted procedures and column diameters for D and AB pollen. Further, we used the same packed columns for all probe experiments with D or AB pollen, respectively. The standard deviations on *k* values in Table 2 are within 1% of the mean value, which is an indication of the absence of irregular voids that affect reproducibility.

**Table 2:** Capacity Factors of Probes on AB Pollen from 30 to 50 °C

probe	30 °C	40 °C	50 °C
acetone	5.86 ± 0.05	4.35 ± 0.04	3.34 ± 0.03
benzene	1.08 ± 0.01	1.02 ± 0.01	0.89 ± 0.01
ethanol	32.6 ± 0.3	24.1 ± 0.2	15.8 ± 0.1
ethyl acetate	2.58 ± 0.02	2.02 ± 0.02	1.62 ± 0.02
isopropanol	16.8 ± 0.2	13.1 ± 0.03	8.41 ± 0.08
pyridine	50.3 ± 0.5	32.0 ± 0.3	21.4 ± 0.2
tetrahydrofuran	2.46 ± 0.02	1.97 ± 0.02	1.60 ± 0.01

Table S2 shows the *A*_S_ values of the probes on AB pollen at three different temperatures. Coupled with increased retention with AB pollen is a significant increase in the *A*_S_ values with increasing polarity. In contrast to D pollen, the *A*_S_ values for the solvent peak on AB pollen were close to ideal values at 1.02 and 0.99. This observation is consistent with the elimination of intine and intracellular material and exposure of significantly more surface area, observed above. Generally, the trends in *A*_S_ with the molecular probe chemical structure parallel the observations made for the capacity factor above. The least polar probe, benzene, had *A*_S_ values of 1.06–1.15, close to cyclohexane, and pyridine, and in most cases, it had the largest *A*_S_ values, ranging from 3.27–4.52. *A*_S_ values of alcohols, acetone, ethyl acetate, and tetrahydrofuran were usually observed to be intermediate between those of benzene and pyridine. These trends are similar to those reported by others for normal phase liquid chromatography from nonpolar carrier solvent onto polar solid phases. For example, pyridine elution from 99% hexane/1% *i*-propanol onto zirconia showed a large, asymmetric tail, whereas benzene was symmetrical, similar to what is observed here.^38^

Figure 5 shows van’t Hoff plots that were used to determine heats of interaction (Δ*H*) for transfer of probes from the mobile phase to the AB pollen surface according to eq 1

1 $$\ln\left(k\right)=-\frac{\Delta H}{RT}+\frac{\Delta S}{R}+\ln\left(\frac{V_{s}}{V_{0}}\right)$$

**Figure 5:**
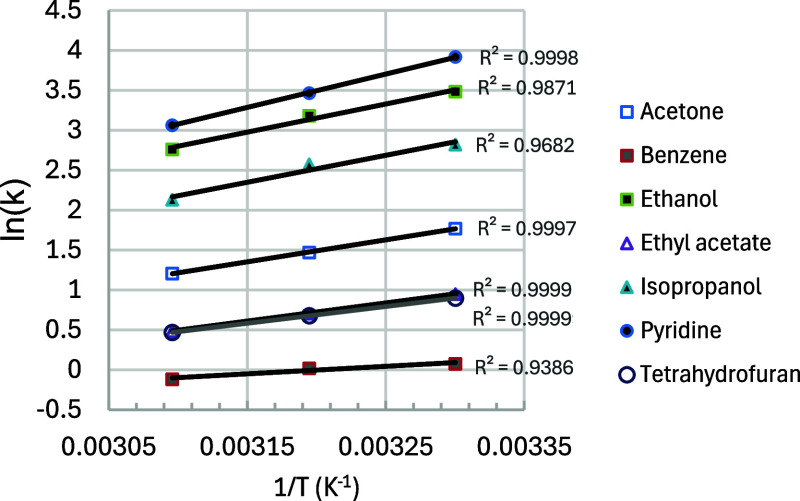
Plots of ln­(*k*) versus 1/*T* (K^–1^) for probes on AB pollen. EA = ethyl acetate. THF = tetrahydrofuran. Error bars on ln­(*k*) are smaller than the symbol size used and thus not shown.

Here, Δ*S* is the entropy for the transfer of the probes from the mobile phase to the stationary phase, *R* is the gas constant, *T* is the absolute temperature *V*_s_ is the volume of the stationary phase, and *V*_0_ is the void volume. The plot shows that ln­(*k*) follows a linear dependence on 1/*T*, with all *r* ^2^ values above 0.92. The heat of interaction values, determined from linear regression, ranged from −9.2 kJ/mol (benzene) to −40.6 kJ/mol (pyridine) (Table 3), increasing monotonically with the probe *k* values. To gain some understanding of the meaning of AB pollen Δ*H* values, we can compare them to enthalpies reported for liquid phase transfer of solutes from nonpolar solvents to polar solids like silica or zirconia. For example, the Δ*H* for benzene adsorption onto zirconia from a hexane mobile phase was reported to be −8.4 kJ/mol,^28^ similar to the result for benzene onto AB pollen from cyclohexane reported here. Silica is a versatile solid phase whose surface chemistry can be tuned to include hydrogen bonding, as well as Brönsted acid sites. The Δ*H* for pyridine adsorption from cyclohexane to two types of hydrogen-bonding silanol sites on silica gel, determined from calorimetric titration, was reported to be −22.2 kJ/mol (vicinal SiOH sites) and −52.8 kJ/mol (bridged SiOH sites).^39^ The value Δ*H* for which we report pyridine on AB pollen was intermediate between these at −40.6 kJ/mol. Pyridine adsorption from cyclohexane was also studied on silica modified with Brönsted acid sites (via 12-tungstophosphoric acid (HPW)), and Δ*H* was −117 kJ/mol at 25% HPW content for the Brönsted site.^40^ These comparisons suggest that AB pollen interaction with polar probes like pyridine is dominated by hydrogen bonding sites consistent with the presence of surface hydroxyls, which is consistent with the structure of sporopollenin. The lone electron pair on the nitrogen of pyridine is able to serve as a hydrogen bond acceptor with protic groups of the AB pollen.^41^

**Table 3:** Calculated Heats of Interaction for Probes on AB Pollen

probe	–Δ*H* (kJ/mol)
acetone	26.7 ± 1.0
benzene	9.2 ± 3.1
ethanol	34.4 ± 5.1
ethyl acetate	22.1 ± 0.9
isopropanol	34.1 ± 7.7
pyridine	40.6 ± 1.2
tetrahydrofuran	20.5 ± 0.9

It is useful to consider how *k*, *A*_S_, and Δ*H* also depend on probe molecular properties. Plots of *k* and *A*_S_ versus the probe kinetic diameter, ε, μ, and δ–δ_cyclohexane_ are presented in the Supporting Information. Generally, *k* decreases as the probe diameter increases (Figure S2a), with pyridine being a strong outlier, but *A*_S_ and Δ*H* are not strongly correlated with diameter (Figure S2b,c). On the other hand, the values of *k*, *A*_S_, and Δ*H* each pass through a maximum with ε, μ, and δ–δ_CH_ (CH = cyclohexane) with occasional differences in the shape of the dependence. Figure 6 shows Δ*H* versus δ–δ_CH_, which indicates how the transfer enthalpy varies with the difference in the probe and carrier solvent solubility parameter. This plot shows a clear maximum for pyridine. Values of Δ*H* for ethanol and propanol fall below pyridine, followed by acetone, then ethyl acetate, and tetrahydrofuran (roughly equal). Finally, benzene, despite having a very similar δ–δ_CH_ as tetrahydrofuran, has a >50% lower Δ*H* value. These trends reveal the important roles of oxygen and nitrogen atoms in the probe structure. They indicate that the presence of furan and ketone bonds produces stronger interactions than benzene, followed by the hydroxyl group. The pyridine nitrogen with its lone electron pair produces the strongest interaction, capacity factor, and peak asymmetry.

**Figure 6:**
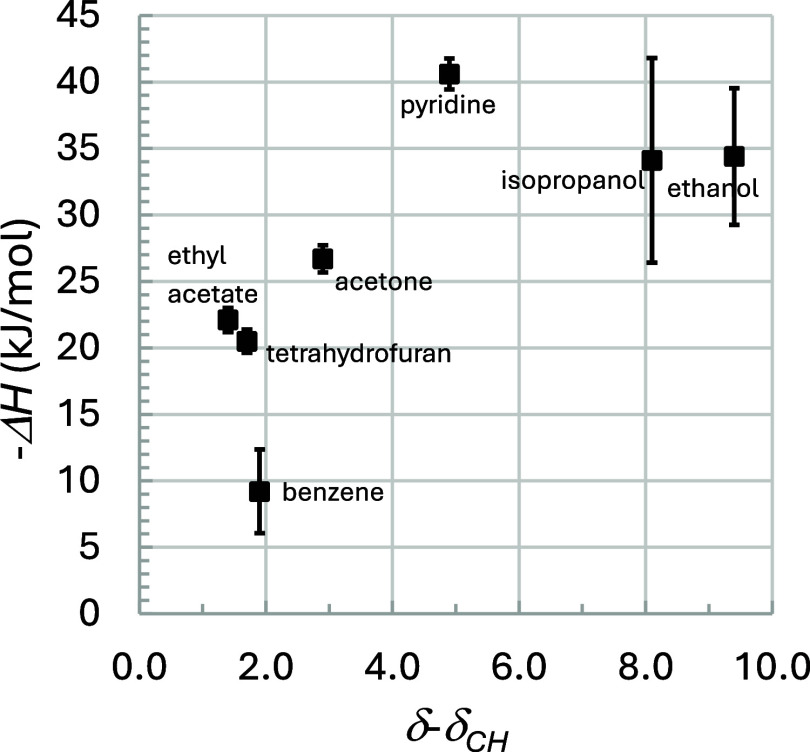
Heat of interaction (−Δ*H*, kJ/mol) of probes on AB pollen as a function of the Hildebrand solubility parameter of the probe relative to the mobile phase (cyclohexane, CH) solubility parameter, δ–δ_CH_, MPa^1/2^.

It is important to note the role that solvent-probe competition for the solid phase may play in the HPLC results. We selected cyclohexane as the carrier solvent as it is nonpolar and has been used as a carrier in other ILC studies of probes with varying polarity.^39,40^ Most probes had similar *k* and *A*_S_ values on D pollen, with ethanol being the major exception (Figure 4). While we attributed this to the amount of pore- and surface-blocking internal cellulose material, we cannot rule out solvent-probe competition as a contributing factor. If competition was the dominant factor in D pollen behavior, however, we would not expect ethanol to have such a pronounced *k* and *A*_S_ compared to isopropanol. We see dramatically different behavior on AB pollen, where there is a very strong dependence of *k*, *A*_S_, and Δ*H* on probe polarity. The enthalpy values represent the transfer between cyclohexane and the pollen solid phase. It is interesting that the Δ*H* of benzene is 50% *lower* than that for tetrahydrofuran, despite their similar δ–δ_CH_. This result may indicate some role of competition between these two hydrophobic species for the AB pollen surface. However, the polarity and specific functional groups of less hydrophobic probes (alcohols, amines, etc.) clearly play a dominant role. We would expect a different ranking of enthalpy values if different carrier solvents were to be used.

We also mention here the potential effects of surface chemical heterogeneity on the interpretation of ILC results presented here. Solid phases can present heterogeneous microenvironments with uneven spatial distribution of functional groups.^42^ Spatial variations in chemical functionality affect the binding site distribution and calculated enthalpies of adsorption. AB pollen is highly porous due to the removal of the intine material. It is possible that the extent of surface modification introduced by the acid treatment is distributed as a function of pore size or depth in the exine wall.^42^ Direct measurement of the chemical group distribution on sporopollenin is beyond the scope of this study. The results presented here represent an average response over all of the pollen chemical microenvironments.

We have utilized ragweed pollen in this study, as it is commonly used in prior studies on pollen chemistry and applications. We consider how results of this study might inform our understanding of the sporopollenin chemistry from other plants. Guilford et al.^8^ analyzed sporopollenin material gathered from a broad phylogenetic range of plant sources (ragweed, wheat, pine, corn). They found that carbon resonances in ^13^C NMR spectra occur in four distinct regions in all species, but they vary in the peak intensity and shape. The results suggest that these sporopollenins are aliphatic polymers that contain ether, hydroxyl, carboxylic acid, ester, and ketone groups. These oxygenated species occur in varying amounts depending on the origin of the sporopollenin used, also indicated in later studies.^1,3–8^ Together, these studies suggest that pollen from different species share qualitatively similar structures with species-specific quantitative variation in composition. Our group has also carried out extensive atomic force microscopy studies of pollen adhesion across families (including ragweed, olive, sunflower, poplar, and dandelion).^43^ The results showed that the effects of chemical differences on adhesion are much less pronounced than the physical effects of the sporopollenin surface morphology. This lends confidence to the expectation that qualitative trends observed for ragweed will also be valid for other species, given the expectation that quantitative differences in retention or Δ*H* are likely.

The solid–liquid interactions revealed by ILC may illuminate our understanding of the compatibility of pollen with other materials in applications such as pollen-polymer composites. The data suggest that polymers with alcohol, amine, or amide groups may have favorable energetic interactions with the AB pollen. In one previous study, D pollen was incorporated in both polystyrene (PS) and polycaprolactone (PCL) to create pollen-polymer composites.^11^ SEM micrographs revealed large voids at the pollen-polymer interfaces, indicating poor adhesion. In the present work, D pollen showed low retention for all probes considered, likely related to difficulty accessing pore surfaces. Our results suggest that AB pollen would be more compatible than D with polymers that possess polar groups such as esters, protic groups like OH, NH and NH_2_, and nitrogen electron lone pairs. Examples include poly­(vinyl acetate) (PVAc), poly­(vinyl alcohol), poly­(vinylpyrrolidone), epoxy, and poly­(urethanes). One prior study compared the interface morphology and mechanical properties of PVAc filled with either D or AB ragweed pollen. Composites with D pollen had poor adhesion between PVAc and pollen, and mechanical properties were degraded relative to neat PVAc.^10^ On the other hand, composites prepared with AB pollen displayed stronger interfacial adhesion. The modulus increased by 29% with an AB pollen loading. Another study reported the preparation of D and AB pollen composites with epoxy polymers formed from epoxide and diamine monomers.^9^ Composites prepared with D pollen displayed weakened mechanical properties. AB pollen exhibited an increase in both tensile strength (by 47%) and strain at break (by 70%), which is indicative of strong polymer-pollen adhesion. The results with PVAc and epoxy polymers correlate with the observations made here for enhanced retention of ester and protic probes on AB versus D pollen. Beyond mechanical properties, these studies connect D and AB pollen surface functionality to their dispersion in polymer films and casting solvents. For example, in ethanol solutions with PVAc, D pollen settles out of the solution in a matter of days. However, AB pollen remains well dispersed in these solution for several months.^10^ We find similar results in suspending D and AB pollen within waterborne epoxide formulations.^9^ Because the time scale for settling of even D pollen was on the order of days and film casting occurs over hours, the dispersion of D pollen in the polymer films was similar to AB pollen.

One can also consider how these results might impact the understanding of the current and emerging applications of sporopollenin in microgels^13^ and as vehicles for drug^14,16^ or vaccine delivery.^15^ Because the internal cellulose and cellular contents of sporopollenin are usually thoroughly removed during preparatory steps in these applications, we consider the results of the AB treatment. We acknowledge that the specific AB treatment utilized here is sometimes not carried out for pharmaceutical applications in lieu of other base or acid treatments. Active pharmaceuticals range from hydrophobic to hydrophilic and often contain polar and Lewis basic groups. Thus, we expect the effects of Lewis acidic sporopollenin surface chemistry illuminated here to influence drug loading and delivery steps. We must be cautious in drawing conclusions because many pollen microgel, drug, and vaccine preparations occur in aqueous media. This introduces solubility and electrostatic effects^44^ that are significantly different than solution characteristics in cyclohexane. Often drug and vaccine formulations contain additional components, e.g., matrices and excipients, which could alter the relevance of our results. Additionally, delivery sites in the human body will typically also be in complex multicomponent aqueous solutions (e.g., mucus membranes). With this caution in mind, we would expect that more polar Lewis base solutes would still have more pronounced interactions with sporopollenin than hydrophobic ones. However, the influence of aqueous solubility could easily outweigh the effects of specific interactions on drug loading or release. For example, hydrophobic species may partition more strongly from water than was observed here in cyclohexane. Hydrophilic species may likewise be more weakly retained from water than from cyclohexane. Conversely, we would expect the opposite trends during release, where water solubility may dominate partitioning.

## Conclusions

Solid–liquid interactions between short ragweed pollen and a series of chemical probes with varying polarities and functionality were measured with ILC in order to further understand sporopollenin surface chemistry. Retention of probes was measured on D and AB pollen over a range of temperatures, allowing the enthalpies of the interaction to be calculated. D pollen displayed similar and low retention and capacity factors for most probes with the exception of ethanol, which was the most polar and smallest probe. This result was correlated with the presence of significant pore-filling residue and retained intine present on D pollen. The capacity factors of probes on AB pollen, which displayed open pores with increased surface area compared with D pollen, depended strongly on probe properties such as dipole moment, presence of protic groups or lone electron pairs, and other factors. The capacity factor, peak asymmetry, and enthalpy of interaction of AB pollen were the lowest for benzene and maximum for pyridine, followed closely by ethanol and *i*-propanol. This result further correlates with the understood chemistry of sporopollenin as possessing abundant ether, ester, and protic groups. The information gained from ILC helps rationalize the behavior of D versus AB pollen when it is used in applications requiring favorable interactions with other molecules, such as polymer composites.

## Experimental Methods

### Materials

Short ragweed (*A. artemisiifolia*) D pollen grains were obtained from Greer Laboratories. HPLC grade acetone, benzene, cyclohexane, ethanol, ethyl acetate (EA), pyridine, and tetrahydrofuran (THF) were obtained from Sigma-Aldrich, and isopropanol was obtained from Alfa Aesar. Selected physical properties of the solvent and probe molecules are presented in Table 1. Kinetic diameters were taken from the literature, data for which were available for benzene,^45^ cyclohexane,^45^ ethanol,^46^ pyridine,^47^ and isopropanol.^46^ Diameters for acetone, ethyl acetate, and tetrahydrofuran were estimated by the correlation 0.841­(*V*_c_)^1/3^, where *V*_c_ is the critical volume in cm^3^/mol.^48^ The dielectric constants (ε) and dipole moments (μ) were obtained from Linstrom and Mallard^49^, and solubility parameters (δ) were obtained from Barton.^50^

### Pollen Preparation

Ragweed was stored at 4 °C prior to use. Experiments in this paper use D pollen grains as received, as well as pollen grains that have been treated by an acid base hydrolysis procedure that dissolves intracellular material and isolate the exine shell.^3,18^ To facilitate comparison to prior literature on pollen composites, we used an AB treatment identical to those studies.^9,10,51^ While extraction of sporopollenin shells is well established in the literature,^3,18^ we optimized the procedures for treatment of larger batches of pollen (10 g or more) to obtain intact pollen grains free of debris, as described previously.^51^ Obtaining intact grains was essential in achieving reproducible column packing, and key variables optimized were the stirring rate, centrifugation steps, and moderate temperature used in the base-hydrolysis step, in particular. Typically, 10 g of pollen were dispersed in 80 mL of DI water in a 500 mL flask. Six grams of KOH were dissolved in 20 mL of water and added to the pollen-water mixture to make a 6 w/v % KOH solution. This was gently stirred for 24 h at room temperature. The mixture was neutralized with HCl and subsequently washed with hot water and hot ethanol, with centrifugation between each step at 2800 rpm. The pollen was then dried in a convection oven at 60 °C overnight. The dried base-hydrolyzed grains were then resuspended in water, sonicated to detach debris, and centrifuged at 2800 rpm. The grains were added to a 500 mL flask while still suspended in a small amount of water. 200 mL of 85% H_3_PO_4_ was added to this flask and was refluxed at 50 °C for 7 days again with gentle stirring. The acid-treated pollen was neutralized with HCl as described previously, followed by washing sequentially with hot water, acetone, and ethanol, centrifugation in between each of these washing steps at 2800 rpm. The washed pollen was then dried in a convection oven at 60 °C.

### Characterization and ILC Experiments

FT-IR spectra were obtained on a Bruker Vertex 80 V FT-IR spectrometer equipped with a MIR beamsplitter from 4000 cm^–1^ to 400 cm^–1^. D and AB pollen were mixed with KBr powders and pressed in pellets for measurement. SEM was performed with a Zeiss Ultra-60 FE-SEM instrument. The specific surface area of pollen was determined on a QuadraSorb SI instrument based on N_2_ adsorption at 77 K by the BET method. D pollen samples were washed rigorously in cyclohexane before commencing ILC experiments and then heated in a vacuum oven at 100 °C before being packed in the column to remove physisorbed water. D and AB pollens were each wet packed with cyclohexane in a stainless-steel column (i.d. of 4.6 mm, 5 cm long, and with 2 μm frits) with the aid of mechanical vibration and a vacuum pump. Cyclohexane was used as the mobile phase. ILC experiments were conducted with a Dionex Ultimate 3000 UHPLC instrument equipped with a UV detector with a flow rate of 0.5 mL/min at various temperatures (30, 40, and 50 °C). The UV detector was operated at a wavelength of 200 nm. 1 to 10 μL injections were made from 0.1 or 1 vol % solutions to adjust for UV absorption signal strength within the dilute concentration regime. Adsorption isotherms were collected for selected probes to verify the range of probe concentration in the linear adsorption regime (Figure S1). As a result, probe concentrations below 1 × 10^–5^ mol/mL were used. Experiments with molecular probes were initiated only after ensuring that no UV signal was detected while flowing only neat carrier solvent, to minimize interference from any possible substances being extracted from the pollen. The dead volume was determined by performing injections with cylcohexane into a dry column, representing an estimate of the void time of an unretained probe, *t*_0_. For the model probes, the retention by the solid phase pollen is described by the capacity factor, *k*, as shown in eq 2

2 $$k=\frac{t_{R}-t_{0}}{t_{0}}$$

where *t*_R_ is the retention time of an adsorbing probe measured at the maximum in peak intensity. Peak asymmetry factors, *A*_S_, are determined by the ratio of the *t*_0.1_ > *t*_R_ peak width at 10% of the maximum to the *t*_0.1_ < *t*_R_ peak width. Error bars presented in the paper are standard deviations derived from data based on three replicates. For enthalpy values, we estimated error by calculating the minimum and maximum values of the slope of van’t Hoff plots based on the extremes in ln­(*k*_avg_) ± its standard deviation.

## Supplementary Material



## Notes

Asymmetry values of D and AB pollen, sample adsorption isotherms, plots of *k*, *A*_S_ and Δ*H* versus probe molecular parameters (PDF)

## †

Sealed Air Corporation, 2415 Cascade Pointe Blvd, Charlotte, NC 20208
