# Supplementary material for: Sporopollenin Surface Characterization with Inverse Liquid Chromatography
Source: ACS Omega. 2025 Dec 8;10(50):61670–8. doi: 10.1021/acsomega.5c07819 (PMC12750199; doi:10.1021/acsomega.5c07819)
Supplement: Supplementary file 1 [file ao5c07819_si_001.pdf]

Supporting Information

Sporopollenin Surface Characterization with Inverse Liquid Chromatography

Oluwatimilehin O. Fadiran[a], J. Carson Meredith[a*]

[a]*School of Chemical and Biomolecular Engineering, Georgia Institute of Technology, Atlanta, GA, 30032, USA*

Table S1. Peak asymmetry A_S of carrier solvent and probes on D pollen.

Probe	30 °C	40 °C	50 °C
Acetone	2.00 ± 0.07	2.52 ± 0.09	3.43 ± 0.12
Benzene	1.79 ± 0.06	1.82 ± 0.06	1.84 ± 0.06
Cyclohexane (solvent)	1.68 ± 0.06	1.72 ± 0.06	1.67 ± 0.06
Ethanol	1.79 ± 0.06	1.81 ± 0.06	1.80 ± 0.06
Ethyl acetate	2.61 ± 0.09	1.86 ± 0.06	2.00 ± 0.06
Isopropanol	2.15 ± 0.07	2.28 ± 0.08	2.68 ± 0.07
Pyridine	2.63 ± 0.09	3.21 ± 0.11	4.04 ± 0.14
Tetrahydrofuran	1.67 ± 0.06	1.68 ± 0.06	1.71 ± 0.06

Table S2. Peak asymmetry of probes on AB pollen.

Probe	30C	40C	50C
Acetone	3.04 ± 0.10	2.94 ± 0.10	3.31 ± 0.11
Benzene	1.15 ± 0.04	1.1 ± 0.04	1.06 ± 0.01
Cyclohexane	1.02 ± 0.03	0.99 ± 0.04	0.99 ± 0.03
Ethanol	-	3.20 ± 0.11	3.59 ± 0.03
Ethyl acetate	2.34 ± 0.08	1.9 ± 0.07	1.88 ± 0.09
Isopropanol	2.56 ± 0.09	3.08 ± 0.11	4.52 ± 0.14
Pyridine	3.27 ± 0.11	4.51 ± 0.16	4.42 ± 0.15
Tetrahydrofuran	2.13 ± 0.07	1.88 ± 0.06	1.84 ± 0.02

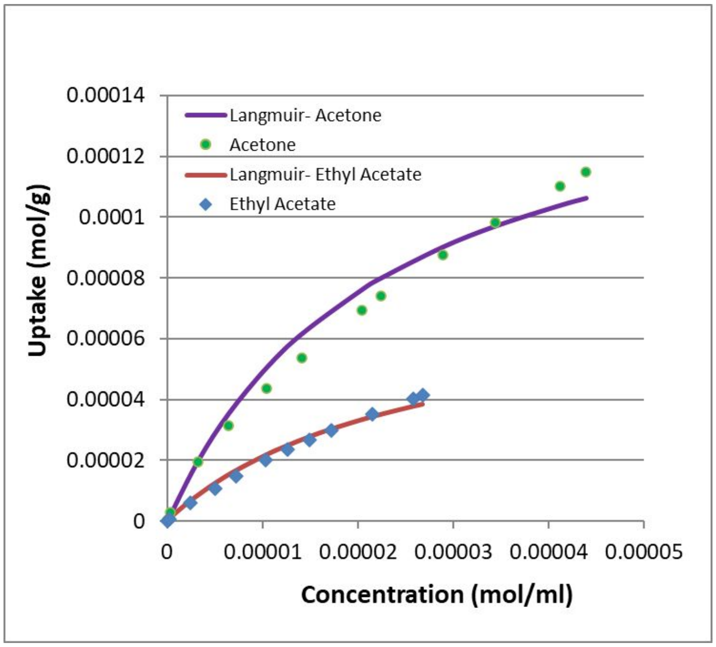

Figure S1. Examples of uptake versus concentration for acetone and ethyl acetate on AB pollen at 30 °C, with Langmuir fitted curves for reference.

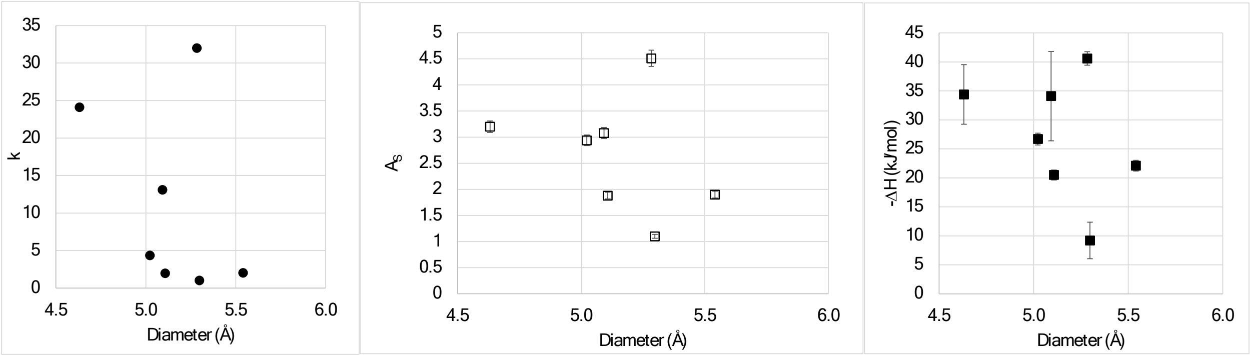

Figure S2. Capacity factor (*k*), peak asymmetry (A_S) and heat of interaction ($-\Delta H$, kJ/mol) of AB pollen as a function of probe kinetic diameter, Å. Error bars for *k* are no larger than the size of the symbol used.

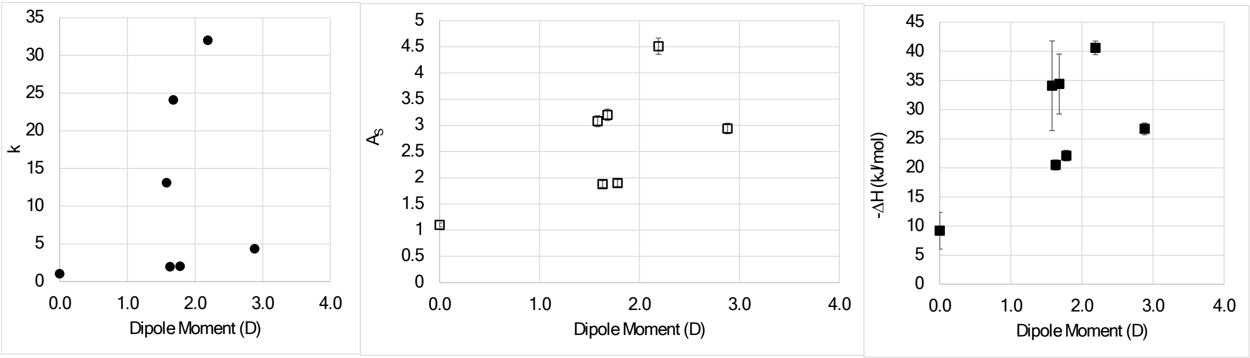

Figure S3. Capacity factor (*k*), peak asymmetry (A_S) and heat of interaction (-ΔH, kJ/mol) of AB pollen as a function of probe dipole moment, Debye (D). Error bars for *k* are no larger than the size of the symbol used.

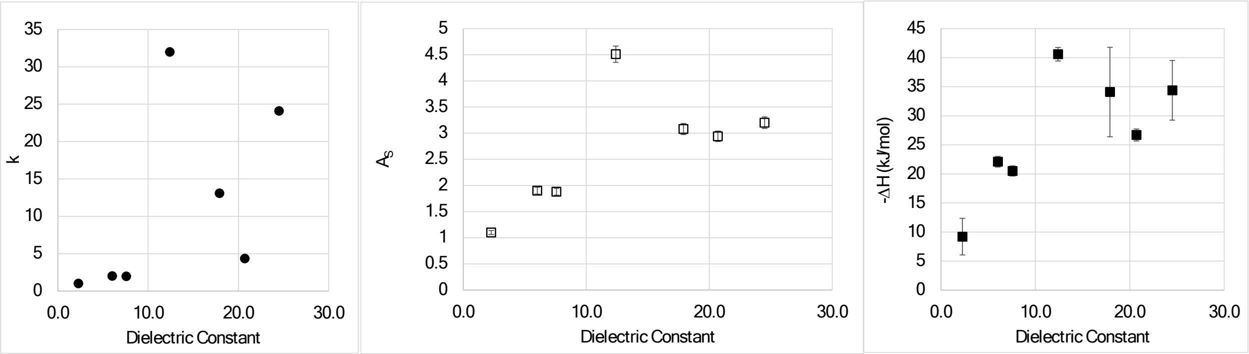

Figure S4. Capacity factor (*k*), peak asymmetry (A_S) and heat of interaction (-ΔH, kJ/mol) of AB pollen as a function of probe dielectric constant. Error bars for *k* are no larger than the size of the symbol used.

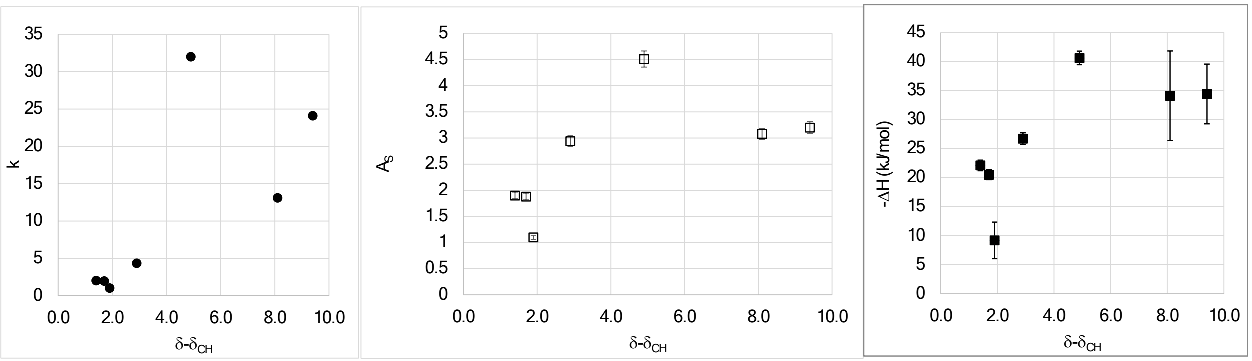

Figure S5. Capacity factor (*k*), peak asymmetry (A_S) and heat of interaction (-ΔH, kJ/mol) of AB pollen as a function of the Hildebrand solubility parameter of probes relative to the mobile phase (cyclohexane, CH) solubility parameter, δ-δ_{CH}, $MPa^{1/2}$. Error bars for *k* are no larger than the size of the symbol used.
